# Study on the mechanism of liver cancer immune escape mediated by MINDY1 through regulation of PD-L1 ubiquitination level

**DOI:** 10.17305/bb.2024.10962

**Published:** 2024-08-31

**Authors:** Xingchao Song, Wenjin Li, Chunyan Tian, Xiao Ma, Weibin Yang, JiaHua Zhou

**Affiliations:** 1 Hepatobiliopancreatic Center, The Affiliated Zhongda Hospital, School of Medicine, Southeast University, Nanjing, China; 2 Department of Hepatobiliopancreatic Surgery, Xuzhou First People’s Hospital, Xuzhou, China

**Keywords:** Motif interacting with ubiquitin-containing novel DUB family-1 (MINDY1), programmed death ligand-1 (PD-L1), ubiquitination, hepatocellular carcinoma (HCC), immune escape, mechanism

## Abstract

The novel deubiquitinase enzyme, motif interacting with ubiquitin-containing novel DUB family-1 (MINDY1), is highly expressed in liver cancer tissues and plays a crucial role in maintaining the stemness of liver cancer cells. Programmed death ligand-1 (PD-L1) is an immunosuppressive molecule overexpressed by tumor cells. The potential role of MINDY1 in inhibiting the stemness of liver cancer cells by deubiquitinating PD-L1 has not yet been reported. To investigate the mechanism by which MINDY1 mediates immune escape in liver cancer through the regulation of PD-L1 ubiquitination, we examined the expression levels of MINDY1 and PD-L1 in liver cancer and adjacent tissues from 50 hepatocellular carcinoma (HCC) patients using protein imprinting and immunohistochemistry. We analyzed the relationship between the expression levels of MINDY1 and PD-L1 in liver cancer tissues and their correlation with the 5-year tumor-free survival rates of patients. Subsequently, MINDY1 expression was knocked down in Huh7 cells using small interfering RNA interference or upregulated through transfection with a MINDY1 overexpression plasmid. The effects of MINDY1 knockdown or overexpression on the proliferation, apoptosis, migration, and invasion of HCC cells, as well as the regulation of PD-L1 binding and ubiquitination, were assessed. The 5-year tumor-free survival rates were significantly lower in both the high MINDY1 expression group and the high PD-L1 expression group (*χ^2^* ═ 4.919 and 13.158, respectively). A significant difference in survival was observed between the high and low MINDY1 expression groups (*χ^2^* ═ 27.415). MINDY1 was found to directly interact with PD-L1, with MINDY1 gene knockdown promoting PD-L1 ubiquitination and MINDY1 overexpression inhibiting PD-L1 ubiquitination. All comparisons yielded statistically significant results (*P* < 0.05). In conclusion, MINDY1 inhibits the malignant progression of liver cancer by inhibiting PD-L1 ubiquitination and mediating immune escape.

## Introduction

Primary liver cancer is a highly prevalent and dangerous malignant tumor in China, with the fifth-highest incidence and the second-highest mortality [1]. The major causes of primary liver cancer are hepatitis B, hepatitis C, and cirrhosis caused by alcohol, followed by aflatoxin, non-alcoholic fatty liver disease, and liver fluke [2]. Hepatocellular carcinoma (HCC) is the most important type of primary liver cancer, accounting for 80% of total cases [3]. HCC has an insidious onset, strong invasion, and a tendency for easy metastasis and recurrence [4]. Treatment of this disease includes surgical resection, transhepatic arterial embolization chemotherapy, molecular targeted drug therapy, immunotherapy, etc., but the results are still unsatisfactory [5]. The progression of HCC in the body is a multi-step process, including occurrence, development, invasion, and metastasis, and involves many factors. At present, the understanding of its molecular events in the medical community is incomplete. Exploring the mechanisms of invasion and metastasis of HCC and searching for molecular targets are still urgent problems to be solved.

The body’s innate immune system can monitor, recognize, and eliminate mutant tumor cells and inhibit their development. However, some tumor cells can evade attack and destruction by the immune system by changing antigen specificity, secreting inhibitory cytokines, or participating in negative immune regulation, etc., leading to immune escape [6, 7]. Programmed death ligand-1 (PD-L1) is an immunosuppressive molecule overexpressed by tumor cells [8]. Clinical studies have found that high expression of PD-L1 is associated with poor prognosis and shortened survival in liver cancer [9, 10]. Additionally, the reduced expression of PD-L1 can promote the killing effect of NK cells on liver cancer cells [11].

The regulation of PD-L1 expression in tumor cells involves multiple levels, such as genome, transcription, post-transcription, translation, and post-translation. Post-translational modifications, such as glycosylation, ubiquitination, and palmitoylation, affect its expression and localization in tumor cells. Ubiquitination refers to the process by which ubiquitin is attached to the target protein via enzymatic action, while the reverse process of ubiquitination is called deubiquitination [12]. Deubiquitination can lead to the activation or deactivation of carcinogenic pathways [13]. Motif interacting with ubiquitin-containing novel DUB family-1 (MINDY1), a novel deubiquitinating enzyme, is highly expressed in liver cancer tissues and maintains the stemness of liver cancer cells, which is associated with a poor prognosis of liver cancer [14]. However, whether MINDY1 can inhibit the stemness of hepatoma cells by deubiquitinating PD-L1 has not been reported. Therefore, we explored the mechanism of MINDY1 mediating liver cancer immune escape by regulating the level of PD-L1 ubiquitination, to provide a reference for clinical immunotherapy of HCC.

## Materials and methods

### Clinical specimen source, cell line, and main reagents

Liver cancer and adjacent tissues (>5.0 cm away from the tumor margin) were obtained from surgical specimens of 50 HCC patients at the Affiliated Zhongda Hospital. The inclusion criteria for patients were as follows: (1) None of the patients had received radiotherapy or chemotherapy before the tissue samples were collected, and all were confirmed by pathological diagnosis. (2) All patients had complete follow-up data and had provided informed consent for sample collection for this study. Follow-up will be completed by December 2023.

The liver cancer cell lines HepG2, HCCLM3, and Huh7 used in this study were purchased from Shanghai Hongshun Biotechnology Co., Ltd. The differentiation degree of HepG2 is relatively high, and the biotransformation characteristics of metabolic enzymes in the cells are relatively complete, without the need for exogenous activation systems. In drug action-related research, its metabolic enzymes remain stable and do not change with increasing passage times. The biotransformation metabolic enzymes it contains are homologous to normal human liver parenchymal cells. Therefore, this cell line can be used for in vitro liver cell metabolism studies. HCCLM3 cells are a highly spontaneous lung metastatic liver cancer cell line widely used in basic and clinical research on the pathogenesis of human liver cancer, as well as in the screening of anti-tumor drugs. Huh7 is AFP positive and highly differentiated, characterized by HBV negativity and susceptibility to the hepatitis C virus (HCV), and can be used for regulating gene expression mechanisms, metabolism, etc. The reason Huh7 cells are more suitable for co-culture is that they have specific advantages in studying tumor metastasis mechanisms. Firstly, Huh7 cells are widely used in liver cancer research because they can simulate some characteristics of human liver cancer, including cell migration and invasion ability. Secondly, the characteristics of Huh7 cells have great potential in studying the biological properties of tumors.

The MINDY1 overexpression plasmid was purchased from Shanghai Tuoran Biotechnology Co., Ltd. DMEM culture medium and fetal bovine serum (FBS) were purchased from Shanghai Lianshuo Biotechnology Co., Ltd. The Transwell chamber (ECM550) was purchased from Chemicon, USA; RNA extraction reagent Trizol was purchased from Invitrogen, USA; the reverse transcription kit was provided by Wuhan Google Biotechnology Co., Ltd., primary antibody and transfection kit were purchased from Guangzhou Ruibo. The peroxidase kit was purchased from Shanghai Dingguo Biotechnology Co., Ltd.

### Cell correlation quantitative assay

#### Peripheral blood lymphocyte isolation

5 mL of peripheral blood was extracted from non-tumor patients using EDTA anticoagulant tubes. Slowly add blood to the surface of the lymphocyte separation solution (5 mL) contained in a 15 mL centrifuge tube; mix gently and centrifuge (2600 rpm, 30 min, room temperature). The lymphocyte layer (the second milky white layer) is collected with a pipette and transferred to a new centrifuge tube. Add PBS and centrifuge (3000 rpm, 20 min), then discard the supernatant. Add 1 mL erythrocyte lysate, thoroughly blow and mix, and transfer to 1.5 mL EP tubes. Let it rest at room temperature for 10 min and centrifuge again (3000 rpm, 20 min, --20 ^∘^C). The white precipitate is the desired lymphocytes.

#### Liver cancer Huh7 cells were co-cultured with lymphocytes

The growth state of Huh7 cells was observed under a microscope. When the cells adhered to the wall by 80%–90%, the original culture medium was discarded, and the cells were washed with PBS twice. 1 mL of 0.25% Trypsin-EDTA was added for digestion for about 3 min. Trim and centrifuge (1500 rpm, 5 min, room temperature). After rinsing with PBS, the medium of DMEM-RPMI 1640 was thoroughly blown and mixed, and 1 mL of cell suspension was added. When the cell adhesion ratio was about 50%, the original culture medium was discarded. After PBS washing, 4 mL of DMEM-RPMI 1640 medium was added. The peripheral blood lymphocytes obtained above were first blown and mixed with 2 mL of DMEM-RPMI 1640 medium. Then, 1 mL of the cell suspension was added to the Huh7 cell culture bottle, which was the cell co-culture medium of Huh7 cells and peripheral blood lymphocytes from HCC. The control group of Huh7 cells (without lymphocytes) was cultured for 24 h with 50% adherent cells.

#### Immunohistochemical method

We followed the instructions of the immunohistochemical Streptomyces antibiotic protein-peroxidase kit. The presence of tan staining in the cytoplasm was defined as positive gene expression, while the absence of tan staining in the cytoplasm was defined as negative.

#### Western blot

After the cells reached confluence, the culture medium was discarded and cleaned with PBS. Denature buffer lysis solution was added to each well, collected in an EP tube, and placed in a 100 ^∘^C metal bath for 10 min. After cooling, the supernatant was centrifuged (4 ^∘^C, 12,000 r/min, 5 min), and the protein concentration was determined using the BCA method. A 20 µg standard protein sample was transferred to the PVDF membrane through 10% SDS-PAGE gel electrophoresis. The membrane was sealed with 5% skim milk powder for 1 h, washed with TBST, antibodies (diluted 1000 times) were added, and the mixture was incubated in a shaking bed at 4 ^∘^C overnight. After re-cleaning, horseradish peroxidase labeled with a secondary antibody was added (diluted 5000 times) and incubated in a room-temperature shaker for 1.5 h. After cleaning again, the ECL reagent (liquid A: liquid B ═ 1:1) was covered with a PVDF membrane, and the image was developed using a gel imaging system.

### Cell biological behavior detection

#### Cell transfection

Huh7 cells at the logarithmic growth stage were inoculated with 5 × 10^5^ cells/well, cultured in 6-well plates for 24 h to reach 60% confluence, and then transfected with fluid. Transfected cells with empty MINDY1 expression vectors were used as the control group. The expression of MINDY1 in Huh7 cells was knocked down using the small interfering RNA (siRNA) interference technique or upregulated by transfection with a MINDY1 overexpression plasmid, which constituted the MINDY1 knockdown group and MINDY1 overexpression group, respectively.

siRNA interference method: 2 µL transfection reagent ietPRIME and 2 µL si-RNFl25 knockdown sequence were added to 200 µL buffer and mixed thoroughly for 15 min, then added to the medium for gentle mixing for 4 h, followed by the addition of with 2 mL fresh medium. These steps were repeated after 24 h for a second transfection, and cells were collected 48 h later.

Transfection with MINDY1 overexpression plasmid: We added PBS solution to two 10 mL centrifuge tubes. Then, the plasmid was added to one tube, and transfection reagent to the other. Both tubes were combined into a centrifuge tube and mixed well. The transfection reagent was added to the plasmid drop by drop and left to stand for 20 min. The mixture was evenly distributed onto Huh7 cell plates and cultured in incubators. Short hairpin RNA (shRNA) was then used to construct stable cell lines with upregulated and downregulated MINDY1.

#### Transwell invasion

The control group, MINDY1 knockdown group, and MINDY1 overexpression group were selected with two wells each, and the number of migrating and invading HCC cells was detected by Transwell assay. After digestion, the cells were washed with PBS buffer solution and re-suspended in 10 g/L BSA. The cell density was adjusted to 10^5^ cells/mL, and 150 µL of cell suspension was added into the Transwell chamber for culture. After one day, ethanol was added and fixed, and cell invasion was observed under a microscope after staining. Cell migration was detected by a cell scratch test. Cells in each group were inoculated into 18-well plates, and when they reached 90% confluence, three vertical lines were made using a 100 µL pipette tip. The cells were washed with PBS buffer and then observed for one day after adding 0.5% serum medium. Cell migration was observed under a microscope.

#### Cell proliferation assay (CCK-8 method)

With green fluorescent protein (GFP) transfected Huh7 (HUH7-GFP) as control, the CCK-8 method was used to assess the effect of MINDY1 transfected Huh7 (HUH7-MINDY1) on cell proliferation. The ratio of CCK-8 to the medium was 1:10, and the original medium was discarded. The cells were washed with PBS, and then CCK-8-containing medium was added. The cells were incubated at 37 ^∘^C for 2 h, and OD values were monitored continuously at 24, 48, 72, 96, and 120 h after transfection to calculate the cell proliferation rate.

#### Cell apoptosis and cycle distribution were detected by flow cytometry

The cells of each group were propagated in 5-well plates and cultured in 5% CO_2_ at 37 ^∘^C. Afterward, 0.25% trypsin was added for digestion, and the cells were centrifuged at 1000 r/min for 10 min. The cells were washed with PBS buffer, centrifuged again, and then 1 mL of PI dye solution was added and incubated at room temperature in the dark for 60 min. Cell apoptosis and cycle distribution were detected by flow cytometry using specific fluorescence.

#### RNA immunoprecipitation (RIP) experiment

600 µL NP40 lysate was added to the cell precipitate, gently blown and mixed, and placed in an ice bath for 10 min. After the ice bath, the sample was centrifuged at 12,000 rpm at 4 ^∘^C for 10 min, and 550 µL of the supernatant was transferred to a new EP tube. The remaining 50 µL of the supernatant was used for total and frozen at --80 ^∘^C for storage. The EP tube containing 550 µL supernatant was mixed with 20 µL of anti-Flag agar–agar beads or 10 µL of anti-Myc immunomagnetic beads pre-treated with PBST, and incubated in a refrigerator at 4 ^∘^C for 2 h. After centrifugation for 5 min, the supernatant was discarded, and the beads containing the precipitate were collected. NP40 or PBST lotion was added and rotated in the refrigerator at 4 ^∘^C for 2 min, and this process was repeated three times. A 5×SDS-PAGE Loading Buffer (50 µL) was added to the cleaned anti-Flag agar–agar beads or anti-Myc immunomagnetic bead precipitates. At the same time, an appropriate amount of 5×SDS-PAGE Loading Buffer was added to the total stored in the refrigerator at --80 ^∘^C, and the mixture was boiled for 10 min before centrifugation. The SDS-PAGE gel electrophoresis was performed.

#### Ubiquitination experiments

Each Huh7 cell sample was mixed with 200 µL of SDS diluent (NP40 diluted with 10% SDS to 2%) and then placed on ice for 5 min after vortexing (repeated three times). The sample was then boiled for 10 min, followed by adding an appropriate amount of NP40 lysate and cooling in an ice bath for 10 min. It was centrifuged at 12,000 rpm at 4 ^∘^C for 10 min. A total of 50 µL of the supernatant was stored it in the refrigerator at --80 ^∘^C for future use. Additionally, the remaining supernatant was collected in an EP tube, mixed with 10 µL of PBST-treated anti-HA immunomagnetic beads, and incubated with rotation in a refrigerator at 40 ^∘^C overnight. After that, the sample was placed on a magnetic rack for 10 s, the supernatant was discarded, and 500 µL of PBST was added, followed by three washes. An appropriate amount of 5×SDS-PAGE Loading Buffer was added to the immunomagnetic bead precipitate and the total sample. Both were then boiled in boiling water for 10 min and centrifuged again. The supernatant was used for SDS-PAGE gel electrophoresis.

**Figure 1.**
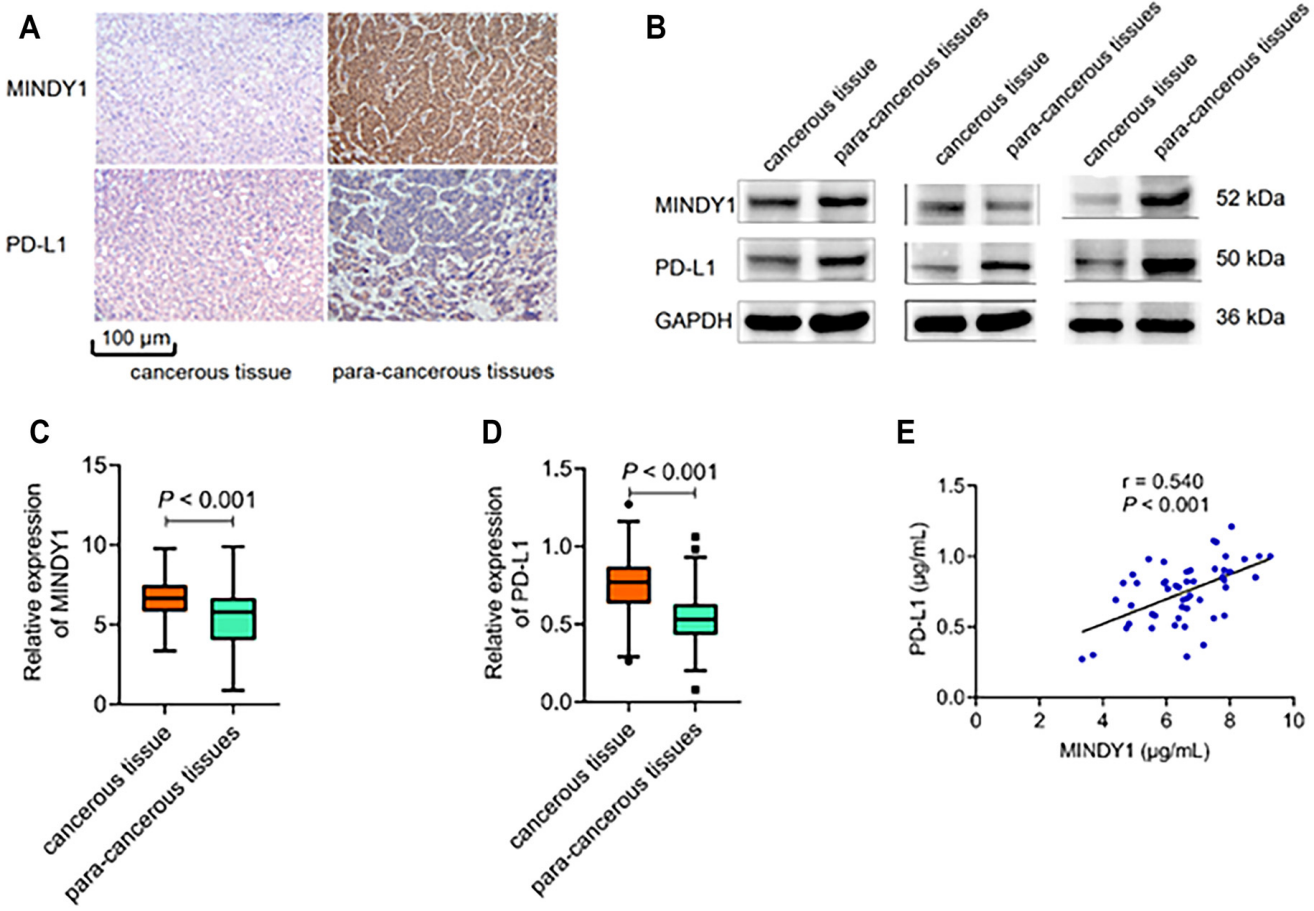
**Expression of MINDY1 and PD-L1.** (A) MINDY1 mRNA extracted from HCC tissues was assessed by immunohistochemical methods; (B) The expression of PD-L1 in HCC tissues was detected by western blot assay; (C) The relative expression of MINDY1 in HCC patients; (D) The relative expression of PD-L1 in HCC patients; (E) Correlation between MINDY1 expression and PD-L1 in cancer tissues. MINDY1: Motif interacting with ubiquitin-containing novel DUB family-1; PD-L1: Programmed death ligand-1; HCC: Hepatocellular carcinoma.

### Ethical statement

This study was approved by the Hepatobiliopancreatic Center, the Affiliated Zhongda Hospital, School of Medicine, Southeast University. Signed informed consent was obtained from every participant.

### Statistical analysis

The experimental data were analyzed using SPSS 23.0 software. Continuous data were described as mean ± standard deviation, and were analyzed using an independent sample *t*-test for normally distributed data. A nonparametric rank-sum test was used for skewed data. Kaplan–Meier curves were used to analyze the influence of gene expression on survival. Pearson correlation was applied to test the correlation between two sets of data. A *P* value < 0.05 showed a significant difference.

## Results

### Expression and correlation analysis of MINDY1 and PD-L1 in different HCC tissues of HCC patients

Immunohistochemical and western blot tests assessed MINDY1 and PD-L1 protein expression in the cancerous and para-cancerous tissues of HCC patients. The results showed that MINDY1 and PD-L1 protein levels in cancerous tissues were higher than those in para-cancerous tissues (Figure 1A and 1B). The relative expression levels of MINDY1 and PD-L1 protein in cancer tissues were 6.56 ± 1.32 µg/mL and 0.75 ± 0.21 µg/mL, respectively, while in para-cancer tissues, they were 5.25 ± 1.83 µg/mL and 0.51 ± 0.18 µg/mL, respectively. The expression levels of MINDY1 and PD-L1 were significantly higher in cancer tissues (*t* ═ 3.949, 5.366; all *P* < 0.001) (Figure 1C and 1D). The expression of MINDY1 in cancer tissues was positively correlated with PD-L1 (*r* ═ 0.540, *P* < 0.001) (Figure 1E).

The average expression values of MINDY1 and PD-L1 in the cancer tissues of HCC patients were divided into high and low-grade groups. Compared with the low expression group (MINDY1 < 6.56 µg/mL or PD-L1 < 0.75 µg/mL), the 5-year tumor-free survival rates were lower in the high expression group of MINDY1 (≥6.56 µg/mL) and the high expression group of PD-L1 (≥0.75 µg/mL) (*χ^2^* ═ 4.919, 13.158; all *P* < 0.05) (Table 1).

**Table 1:** Effects of MINDY1 and PD-L1 on 5-year tumor-free survival in HCC patients

**Group**	**MINDY1 high expression group (*n* ═ 27)**	**MINDY1 low expression group (*n* ═ 23)**	**PD-L1 high expression group (*n* ═ 28)**	**PD-L1 low expression group (*n* ═ 22)**
5-year tumor-free survival	8 (29.63)	14 (60.87)	6 (21.43)	16 (72.73)
Die	19 (70.37)	9 (39.13)	22 (78.57)	6 (27.27)
*χ^2^*	4.919	13.158		
*P*	0.027	0.001		

The numbers represent *n* (%). MINDY1: Motif interacting with ubiquitin-containing novel DUB family-1; PD-L1: Programmed death ligand-1; HCC: Hepatocellular carcinoma.

**Figure 2.**
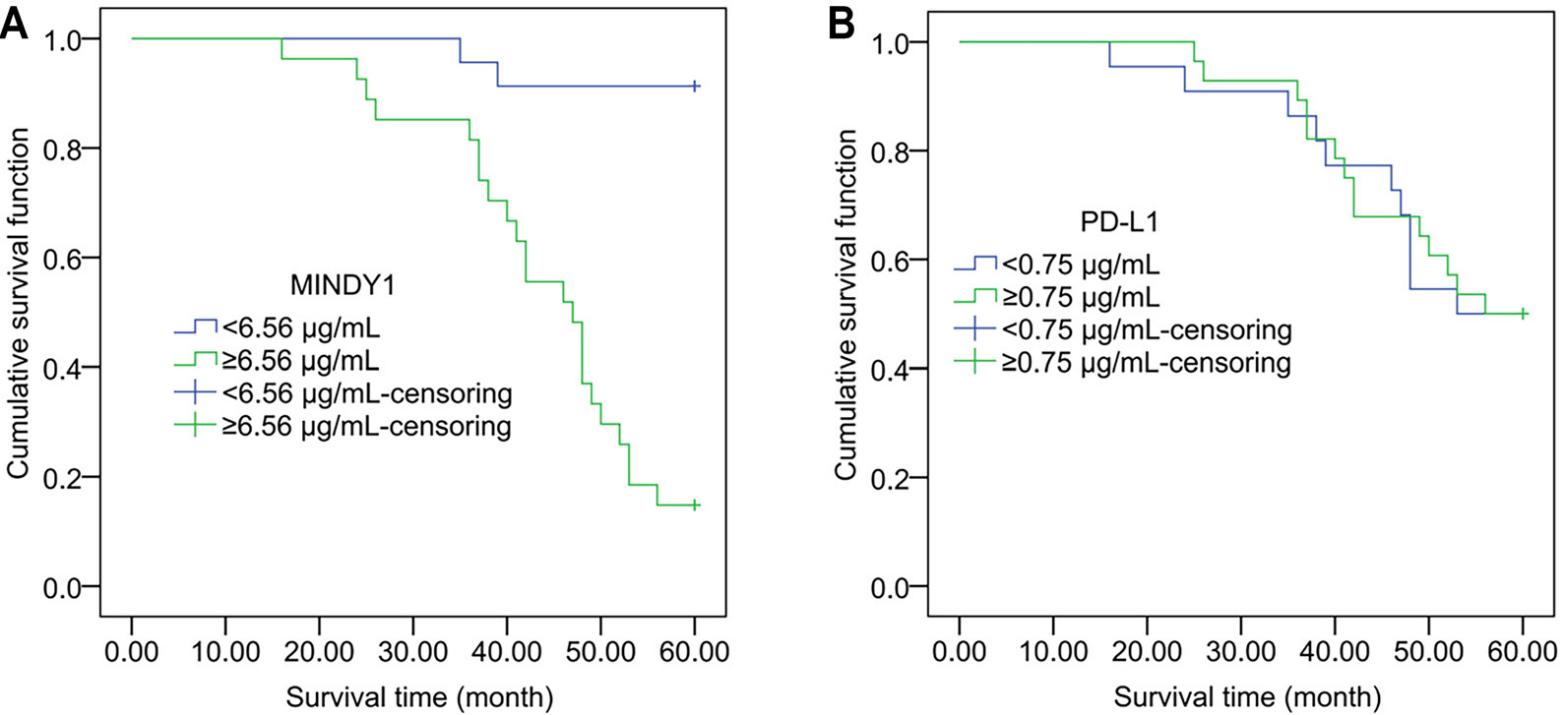
**Effects of MINDY1 and PD-L1 on survival in HCC patients**. Kaplan–Meier curve illustrating the effect of MINDY1 (A) and the effect of PD-L1 (B). MINDY1: Motif interacting with ubiquitin-containing novel DUB family-1; PD-L1: Programmed death ligand-1; HCC: Hepatocellular carcinoma.

Kaplan–Meier curve analysis showed a statistically significant difference in the survival period between the high and low MINDY1 expression groups (*χ^2^* ═ 27.415, *P* < 0.001). However, there was no significant difference between the high and low PD-L1 expression groups (*χ^2^* ═ 0.006, *P* ═ 0.939) (Figure 2).

### The relationship between MINDY1 and PD-L1 in cancer tissues and clinicopathology

Continuous data were categorized by mean value, and the relationship between the expression levels of MINDY1 and PD-L1 in HCC patients and clinicopathological features was analyzed. The results showed no statistically significant differences in the expression levels of MINDY1 and PD-L1 across different ages, sex, HBsAg, AFP, ALT, tumor size, BCLC staging, tumor number, cirrhosis, portal vein tumor thrombi, microvascular invasion, and other basic conditions (*P* > 0.05) (Table 2).

**Table 2:** The relationship between MINDY1 and PD-L1 and clinicopathology in HCC patients

**Data**	**Case number**	**MINDY1 (µg/mL )**	**t/Z**	***P***	**PD-L1 (µg/mL)**	**t/Z**	***P***
Age			--1.086	0.283		--1.617	0.112
≤58 years	27	6.37 ± 1.29			0.70 ± 0.21		
>58 years	23	6.78 ± 1.28			0.80 ± 0.20		
Gender			--0.843^*^	0.399		--1.136	0.261
Male	34	6.44 ± 1.31			0.72 ± 0.21		
Female	16	6.80 ± 1.30			0.80 ± 0.21		
HBsAg			1.041	0.303		0.829	0.411
Positive	38	6.67 ± 1.24			0.76 ± 0.22		
Negative	12	6.21 ± 1.48			0.70 ± 0.20		
AFP			1.724	0.091		1.036	0.306
Positive	41	6.71 ± 1.22			0.76 ± 0.21		
Negative	9	5.88 ± 1.51			0.68 ± 0.20		
ALT			0.132	0.896		--0.872	0.387
≤40 U/L	20	6.59 ± 1.29			0.71 ± 0.20		
>40 U/L	30	6.54 ± 1.33			0.77 ± 0.22		
Tumor size			0.978	0.333		0.902	0.371
≤5 cm		6.78 ± 1.32			0.78 ± 0.24		
>5 cm		6.41 ± 1.29			0.72 ± 0.19		
BCLC staging			1.590	0.118		0.552	0.583
A	29	6.81 ± 1.29			0.76 ± 0.23		
C	21	6.21 ± 1.28			0.73 ± 0.19		
Tumor number			--1.167^*^	0.243		1.282	0.206
Single	36	6.71 ± 1.26			0.77 ± 0.21		
Multiple	14	6.16 ± 1.39			0.68 ± 0.16		
Cirrhosis			--1.493	0.142		0.130	0.897
Yes	35	6.13 ± 0.94			0.75 ± 0.20		
No	15	6.74 ± 1.41			0.74 ± 0.22		
Portal vein cancer thrombi			0.674	0.504		1.399	0.168
Yes	13	6.63 ± 1.32			0.77 ± 0.21		
No	37	6.34 ± 1.28			0.67 ± 0.14		
Microvascular invasion			--1.020^*^	0.308		--0.169	0.866
Yes	20	6.35 ± 1.37			0.74 ± 0.25		
No	30	6.87 ± 1.16			0.75 ± 0.15		

^*^Indicates that a non-parametric rank sum test (*Z*) was used. HBsAg: Hepatitis B surface antigen; AFP: Alpha-fetoprotein; ALT: Alanine aminotransferase; BCLC staging: Barcelona clinic liver cancer staging; MINDY1: Motif interacting with ubiquitin-containing novel DUB family-1; PD-L1: Programmed death ligand-1; HCC: Hepatocellular carcinoma.

### The effects of MINDY1 and PD-L1 on HCC cells and their mechanisms detected at the cellular level

#### Expression of MINDY1 and PD-L1 in HCC cells

**Figure 3.**
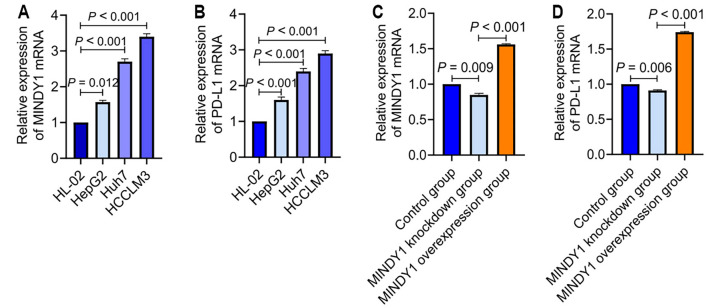
**Expression of MINDY1 and PD-L1 in hepatocellular carcinoma cells**. (A) Relative expression of MINDY1 mRNA; (B) Relative expression of PD-L1 mRNA; (C) Relative expression of MINDY1 in each group; (D) Relative expression of PD-L1 in each group. MINDY1: Motif interacting with ubiquitin-containing novel DUB family-1; PD-L1: Programmed death ligand-1.

The mRNA relative expressions of MINDY1 and PD-L1 in human HCC HepG2, HCCLM3, and Huh7 cells were higher than in human normal liver epithelial cells HL-02. Additionally, the mRNA relative expressions of MINDY1 and PD-L1 in HCCLM3 and Huh7 cells were higher than in HepG2 cells (Figure 3A and 3B). MINDY1 and PD-L1 expressions were increased in the MINDY1 overexpression group, while they were decreased in the MINDY1 knockdown group (Figure 3C and 3D).

#### Effects of knockdown or overexpression of MINDY1 on proliferation, apoptosis, migration, and invasion of HCC cells

**Figure 4.**
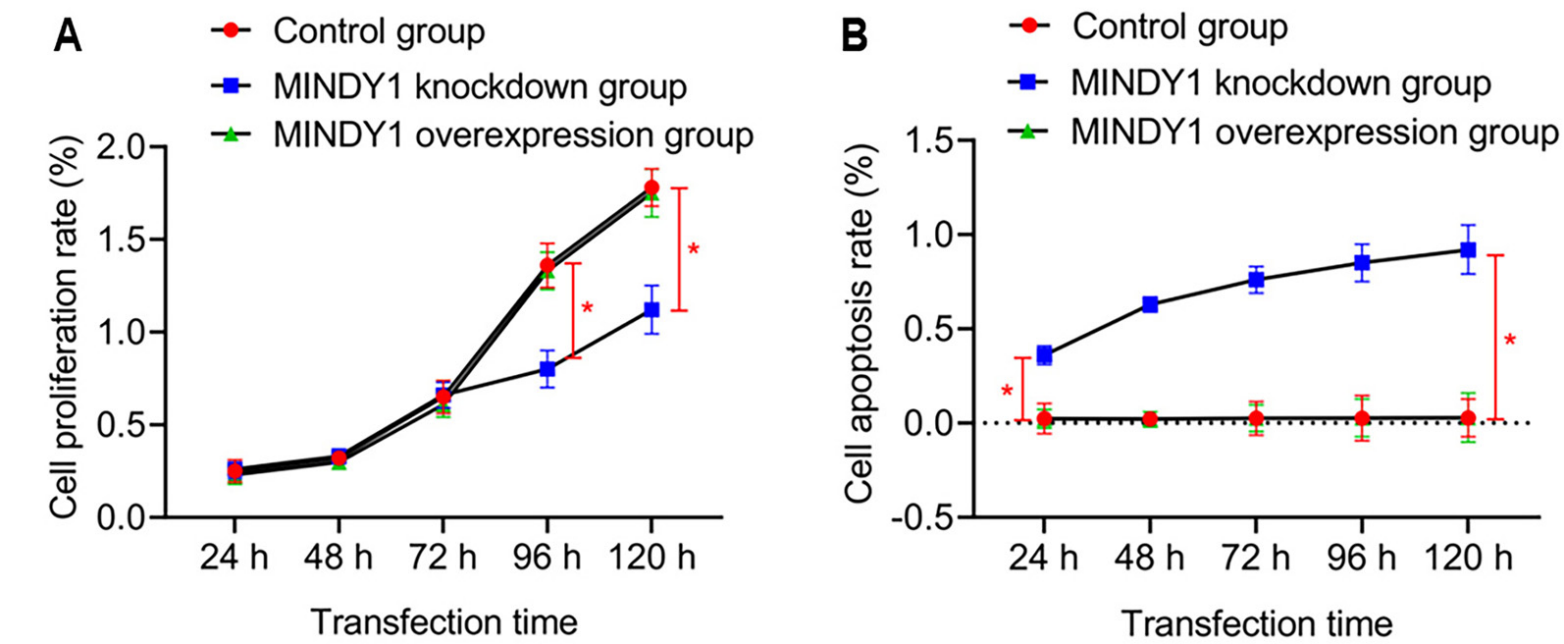
**Proliferation and apoptosis of knockdown or overexpressed MINDY1 cells.** (A) Cell proliferation; (B) Cell apoptosis. ^*^Indicates *P* < 0.05 for comparison between two groups of data. MINDY1: Motif interacting with ubiquitin-containing novel DUB family-1.

Compared with the control group and the MINDY1 overexpression group, the proliferation rate of HCC cells in the MINDY1 knockdown group decreased 72 h after transfection, while the apoptosis rate increased gradually 24 h after transfection (all *P* < 0.05) (Figure 4). Compared with the control group, the number of migratory and invasive cells decreased in the MINDY1 knockdown group, and increased in the MINDY1 overexpression group (*P* < 0.05) (Table 3). Compared with the control and MINDY1 overexpression groups, the proportion of HCC cells in the G1 phase increased in the MINDY1 knockdown group (all *P* < 0.05) (Table 4).

**Table 3:** Transwell cell invasion experiment migration and invasion cell number of Huh7 cells in each group

**Group**	**Migration cell number**	**Invasion cell number**
Control group	436 ± 15	472 ± 20
MINDY1 knockdown group	169 ± 22^a^	178 ± 23^a^
MINDY1 overexpression group	1103 ± 32^a^	925 ± 25^a^

^a^Compared with control group, *P* < 0.05. MINDY1: Motif interacting with ubiquitin-containing novel DUB family-1.

**Table 4:** Cell cycle distribution in each group

**Group**	**G_1_ (%)**	**S (%)**	**G_2_ (%)**
Control group	30.35 ± 3.08	31.22 ± 3.09	32.04 ± 3.06
MINDY1 knockdown group	59.13 ± 5.26^b^	19.16 ± 2.50^b^	19.22 ± 2.48^b^
MINDY1 overexpression group	30.43 ± 3.18	31.58 ± 3.14	32.11 ± 3.12

^b^Compared with control group or MINDY1 knockdown group, *P* < 0.05. MINDY1: Motif interacting with ubiquitin-containing novel DUB family-1.

### Interaction of MINDY1 with PD-L1 and ubiquitination modification

**Figure 5.**
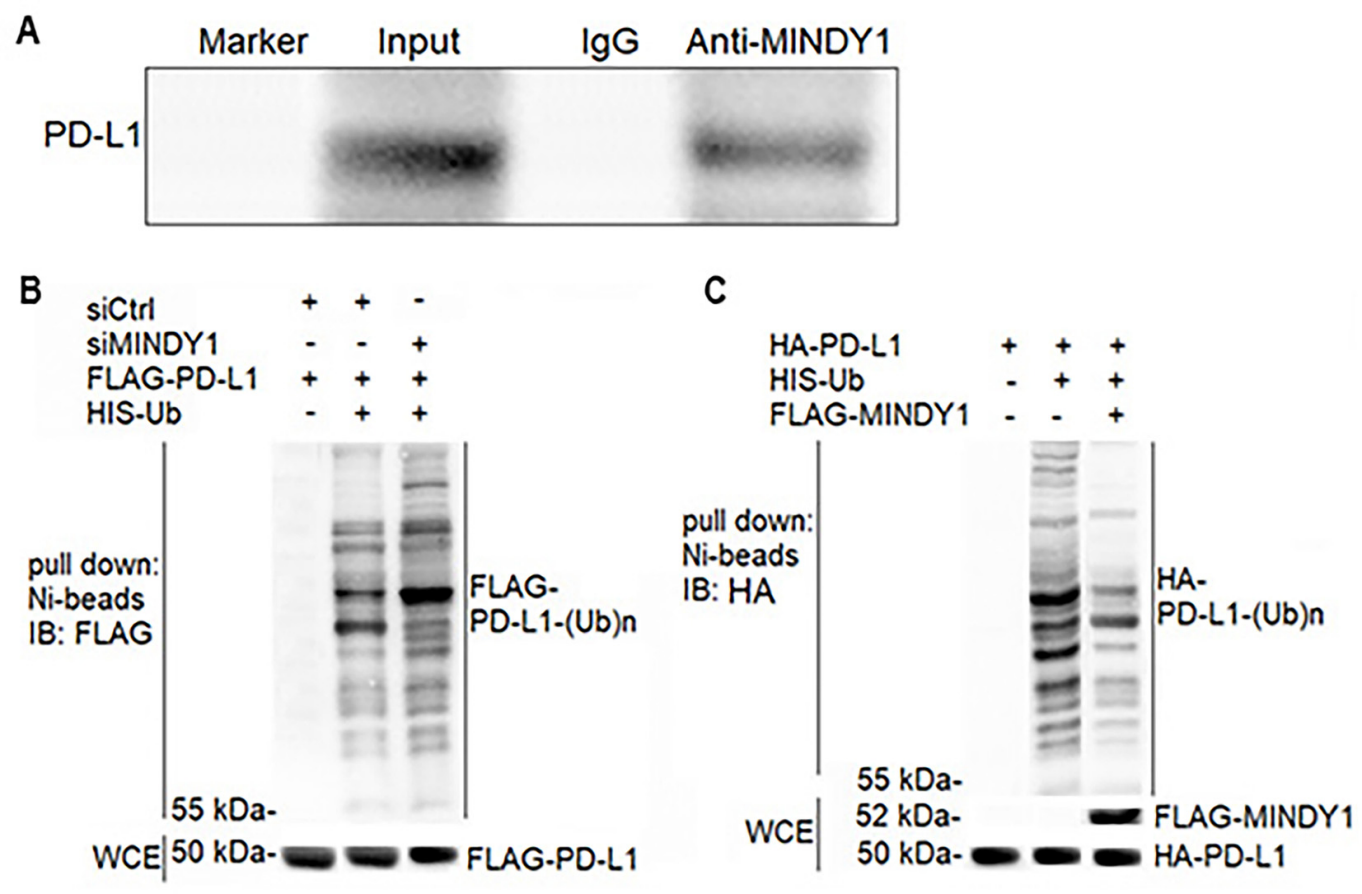
**Interaction between MINDY1 and PD-L1 and the ubiquitination modification detected by RIP experiment.** (A) MINDY1 interacts with PD-L1; (B) PD-L1 ubiquitination during MINDY1 knockdown; (C) PD-L1 ubiquitination during MINDY1 overexpression. MINDY1: Motif interacting with ubiquitin-containing novel DUB family-1; PD-L1: Programmed death ligand-1; RIP: RNA immunoprecipitation.

RIP experiment results showed that anti-Mindy1 antibodies could precipitate PD-L1, indicating that MINDY1 directly interacts with PD-L1 (Figure 5A). The results of ubiquitination experiments indicated that MINDY1 gene knockdown promoted PD-L1 ubiquitination, while MINDY1 gene overexpression inhibited PD-L1 ubiquitination (Figure 5B and 5C).

## Discussion

The microenvironment within an HCC tumor is highly immunosuppressive, and the effector T-cell response is very poor in advanced HCC [15]. Tumor immune escape is one of the main reasons for the further development and deterioration of liver cancer, and overexpression of tumor cell PD-L1 can promote tumor immune escape [16]. PD-1/PD-L1 inhibitors can target the inhibition of the PD-1/PD-L1 signaling pathway and induce the recombination of antigen peptide-histocompatibility complex (MHC) and T cell receptor (TCR), thus activating the anti-tumor immune response of T lymphocytes and inhibiting the growth of tumor cells [17, 18]. The novel deubiquitinase MINDY1 is highly expressed in liver cancer tissues and maintains the stemness of liver cancer cells. It is worth exploring whether it can inhibit the stemness of liver cancer cells by deubiquitinating PD-L1. However, there are few related research reports.

Our study found that the expression levels of MINDY1 and PD-L1 proteins in HCC patients’ cancer tissues were higher than those in paracancerous tissues, suggesting that MINDY1 may be a maintenance factor for HCC cells. The expression of MINDY1 was positively correlated with PD-L1, indicating that the deletion or mutation of the MINDY1 gene might affect the transcription or protein expression of PD-L1 in some way. In terms of patient prognosis, patients with high expression of MINDY1 have a higher 5-year tumor-free survival rate, while those with high expression of PD-L1 have a lower 5-year tumor-free survival rate, which is consistent with previous studies [19]. Additionally, there was a significant difference between the high and low expression of MINDY1 in cancer tissues and the survival time of HCC patients, with patients with high expression of MINDY1 showing a trend of lower survival time. However, we did not find that the expression levels of MINDY1 and PD-L1 were correlated with the clinicopathologic data of HCC patients, which may be related to the small number of samples collected—this is also a limitation of this experiment and may lead to statistical bias regarding pathological features. Many studies have found that high expression of MINDY1 is a risk factor for poor cancer prognosis [20], which is inconsistent with this study. We speculate that it may be related to the high expression of PD-L1 in liver cancer tissues. PD-L1 activity in CD8+ T cells and cancer/immune/stromal cells participates in breast cancer immune escape [21]. Anti-PD-L1 immunotherapy is becoming increasingly important in cancer treatment. The upregulation of PD-L1 expression in cancer cells depends on acidosis and the induction of IFN-γ, a novel immune escape mechanism in response to anti-PD-L1/PD-1 therapy [22]. Predicting patients’ responses to PD-1/PD-L1, monitoring disease progression, and predicting clinical outcomes based on biomarkers is an effective method for guiding cancer immunotherapy [23].

By observing the effects of knocking down or overexpressing MINDY1 on the proliferation, apoptosis, migration, and invasion of liver cancer cells, we can systematically understand the impact and overall effect of changes in MINDY1 expression on various aspects of liver cancer cells. Our results showed that the proliferation rate of MINDY1 knockdown HCC cells decreased 72 h after transfection, and the apoptosis rate gradually increased 24 h after transfection, indicating that MINDY1 knockdown has a certain inhibitory effect on the growth and reproduction of HCC cells and promotes apoptosis. We also found that the number of migrating and invading cells decreased in MINDY1 knockdown HCC cells, while the number increased in the MINDY1 overexpression group. The proportion of MINDY1 knockdown HCC cells in the G1 phase increased. These results indicated that the knockdown of the MINDY1 gene could block the migration, invasion, and cell cycle distribution of HCC cells, thus affecting their continuous development. Changes cell cycle can have certain effects on cell apoptosis and proliferation, which are fundamental aspects of cell behavior [24]. Retarding the cell cycle distribution of cancer cells plays an important role in promoting apoptosis. The cell cycle is divided into G1, S, and G2 phases, which correspond to the early phase, the synthetic phase, and the late phase of DNA synthesis. Blocking liver cancer cells in the G1 phase can enhance their apoptosis capacity, thus inhibiting the proliferation of liver cancer cells [25, 26]. The above research results indicate that knocking down the MINDY1 gene can inhibit the proliferation, migration, and invasion of liver cancer cells, as well as promote their apoptosis. The independent effect of MINDY1 gene knockdown in liver cancer cells was demonstrated.

We conducted further ubiquitination experiments and found that anti-MINDY1 antibodies can precipitate PD-L1, indicating that MINDY1 can directly interact with PD-L1, and regulate the stability of the PD-L1 protein. This further confirms the key role of MINDY1 in the PD-L1 repair pathway. MINDY1 gene knockdown promotes PD-L1 ubiquitination, while MINDY1 gene overexpression inhibits PD-L1 ubiquitination. PD-L1 specifically binds to programmed death 1 (PD-1), promoting the immunoreceptor tyrosine-based inhibition motif (ITIM) and immunoreceptor tyrosine-based switch motif (ITSM) in PD-1 cells, which recruit protein tyrosine phosphatase 1 (SHP-1) and SHP-2. This, in turn, blocks T-cell signal transduction, leading to T-cell dysfunction [27, 28]. T-cell dysfunction severely weakens immune function, promoting the immune escape of tumor cells and thus tumor progression [29, 30]. Xia et al. [31] showed that MINDY1 is an independent risk factor for maintaining stem cell characteristics and poor prognosis in HCC. Their findings revealed that MINDY1 was highly expressed in liver cancer stem cells with expression levels in liver cancer tissues being higher than in adjacent tumors. The growth of transplanted tumors after MINDY1 knockout was significantly reduced and inhibited, consistent with our study results. In addition, Tang et al. [20] suggested that MINDY1 promotes the proliferation of breast cancer cells, and this induction was related to the stabilization of estrogen receptor alpha. Luo et al. [32] also confirmed that MINDY1 plays a catalytic role in the YAP deubiquitination enzyme, thereby promoting the progression of bladder cancer. Although MINDY1 is a deubiquitinase enzyme, it has good specificity for leaving K48-linked polyubiquitin chains [14]. However, little is known about its catalytic mechanism, and more reliable studies are needed to explore it. We hypothesized that after MINDY1 deletion in HCC, PD-L1 expression may be regulated through the ubiquitination modification pathway, thus affecting the immunity of the liver cancer tumor microenvironment. However, there are limitations in this study, namely, the failure to clarify whether MINDY1 is an independent prognostic factor in HCC patients, and to detect the effects of MINDY1 and PD-L1 on tumor growth in mice with liver cancer transplantation. Therefore, more in-depth studies are needed to provide more evidence for clinical studies on the MINDY1 gene and liver cancer immunotherapy. We investigated the immune escape mechanism of liver cancer mediated by MINDY1 by regulating PD-L1 ubiquitination levels at the cellular level, but with limitations. Although we have conducted in-depth research on the immune escape landscape and molecular mechanisms of MINDY1 in liver cancer, there are significant differences in the biological mechanisms and frequencies related to immune escape among different cancer types, which increases the complexity of the study and limits the widespread application of our research results.

## Conclusion

In summary, MINDY1 and PD-L1 mRNA expression levels are positively correlated in liver cancer, and MINDY1 can inhibit PD-L1 ubiquitination to mediate immune escape in liver cancer, thereby preventing the malignant progression of the disease.

## Supplemental data

**Graphical Abstract.**
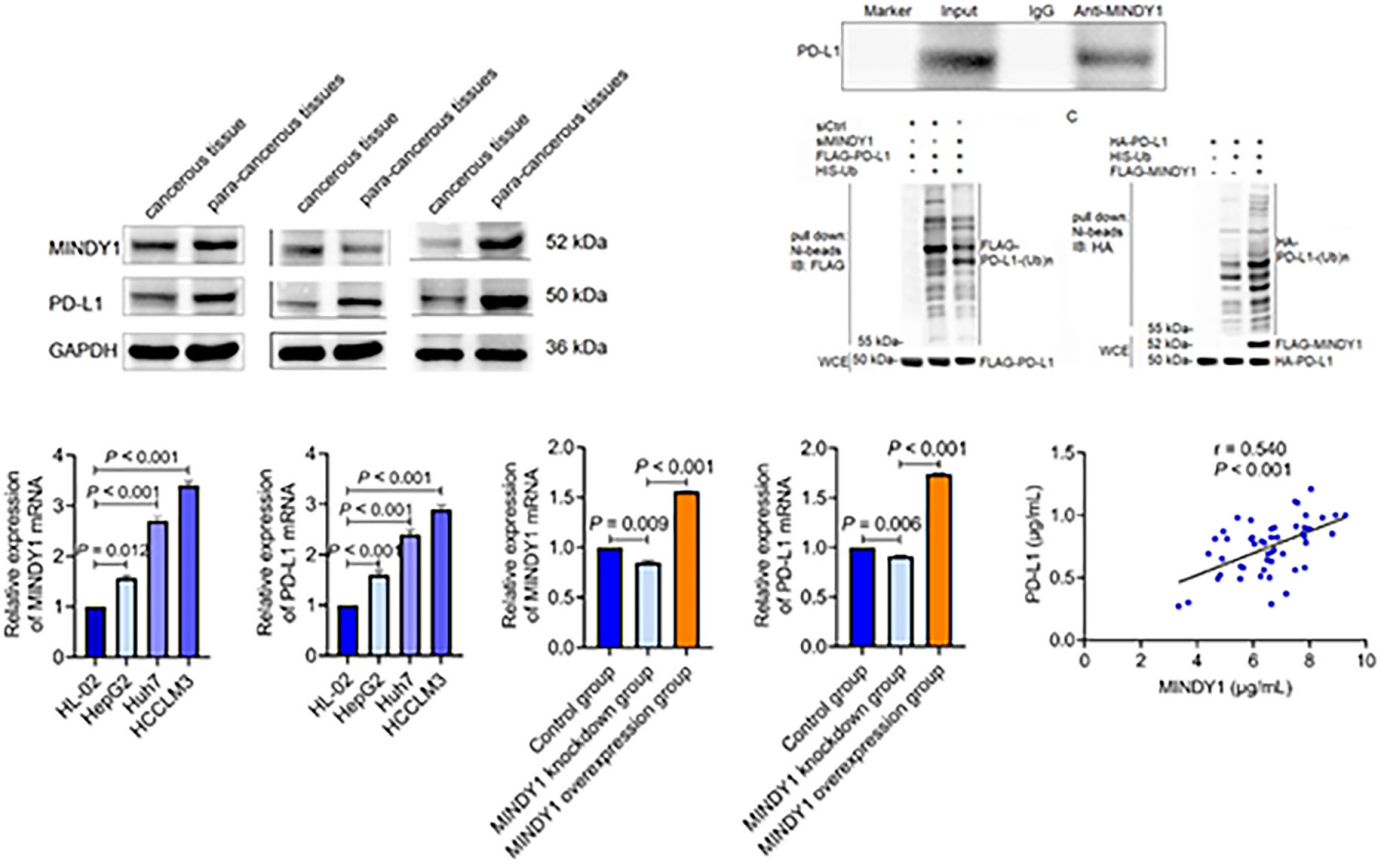
**Study on the mechanism of liver cancer immune escape mediated by MINDY1 through regulation of PD-L1 ubiquitination levels.** We investigated the relationship between the expression levels of MINDY1 and PD-L1 in liver cancer and adjacent tissues and prognosis, and observed the effects of MINDY1 knockdown or overexpression on the proliferation, apoptosis, migration, and invasion of liver cancer cells, as well as the regulation of PD-L1 binding and ubiquitination. The results confirmed a positive correlation between the expression levels of MINDY1 and PD-L1 mRNA in liver cancer. MINDY1 inhibits PD-L1 ubiquitination-mediated immune escape in liver cancer, thereby preventing the malignant progression of liver cancer. This can provide a reference for exploring the mechanisms of HCC invasion and metastasis and identifying molecular targets for liver cancer.

## Data Availability

The corresponding author can provide the data supporting the findings of this study upon request.
